# Stem cells, aging and cancers

**DOI:** 10.18632/aging.100776

**Published:** 2015-07-07

**Authors:** Arnold J. Levine

**Affiliations:** Institute for Advanced Study Princeton, NJ 08540, USA

Recently tissue specific stem cells from mammals have been isolated, characterized and grown in three-dimensional cultures producing faithful organoids composed of all the cell and tissue types of the organ [1]. In all cases of diverse tissue specific stem cells the WNT signal transduction pathway functions to promote replication of the stem cells and is regulated by R-spondins acting upon G-linked receptors, LGR 4, 5 and 6, which prove to be excellent biomarkers for these stem cells [1). John Cairns first pointed out [2] that the stem cells in rapidly renewing tissues of animals with long life spans compete for space and nutrients in their nitches and they will undergo mutations and natural selection for variants that propagate well and have reduced potentials for differentiation leading to the precursors of cancers. Based upon this he proposed that such stem cells required excellent DNA repair processes that reduce the mutation rate to prevent or lower the incidence of cancers. Indeed it has been possible to introduce mutations (APC, RAS, TGF-beta and p53) into normal colon stem cells and reproduce adenocarcinomas of the colon [3]. In support of the stem cell hypothesis as the initiator of cancers p53 mutations have been isolated from clones (derived from stem cells) of epithelial cells of the esophagus in patients with Barrett's esophagus and in hematopoetic stem cells and progenitor cells from patients treated for AML [4]. A second line of support for this idea comes from the observations measuring the number of hematopoetic stem cells in mice, cats or humans [5] and surprisingly the number of these stem cells increases with age (selection of more fit cells for replication) but the ability to transplant these cells into a recipient declines with age (selection of less fit for differentiation). All these results are consistent with the hypothesis that mutations arise in tissue specific stem cells that favor cell division and reduce the probability of commitment to differentiation. What is the nature of these mutations?

In stem cells a splice variant of the p53 protein (delta-40-p53) is produced which, deletes the first 40 amino acids of p53 and the first transactivation domain so that the delta-40- p53 protein fails to transcribe efficiently the genes that full length p53 normally regulates (like p21) [6]. Restoration of the full- length p53 protein and its activation in stem cells stops cell division (via p21) and triggers differentiation of the stem cell [7]. The activation of full- length p53 in stem cells followed by differentiation has been observed in tissue regeneration of planarians and salamanders, as well as in mouse IPS cells and cancerous teratocarcinoma stem cells [7]. In mice with inherited p53 mutations the T-cell lineage stem cell acquires and selects PTEN mutations prior to entry of the stem cell into the thymus and the V-D-J recombination steps and the production of differentiated T-cells [8]. While p53 and possibly PTEN are examples of mutations in stem cells that promote cell division and slow differentiation, we can expect that an entire class of mutations in genes that mediate epigenetic changes should also produce these results. While mutations residing and accumulating in tissue specific stem cells over a lifetime can give rise to cancers, they can also produce abnormalities in tissue functions that contribute to the diseases of the elderly. One might predict a decline in the balance and efficiency of the immune system, abnormal cytokine production, poor metabolic functions and neurodegeneration all could result from mutations that may accumulate in the tissue specific stem cells over a lifetime.

It is ironic that the very body plan of vertebrates employing stem cells to regenerate our tissues, give us longevity and eliminate large numbers of poorly functioning cells through terminal differentiation and cell death relies upon the presence of stem cell populations that compete over a life time to produce the fittest stem cell with a mutational profile that maximizes replication and reduces the efficiency of differentiation. These ideas bring together stem cells, cancers and longevity employing a common evolutionary process acting in both an individual and in populations.
